# Photo Quiz: Unusual phenotypic and Lancefield group testing in blood culture isolate

**DOI:** 10.1128/jcm.01187-23

**Published:** 2024-05-08

**Authors:** Christopher C. Attaway, Erick Tobin, Daniel D. Rhoads

**Affiliations:** 1 Department of Laboratory Medicine, Cleveland Clinic, Cleveland, Ohio, USA; 2 Department of Pathology, Cleveland Clinic Lerner College of Medicine, Case Western Reserve University, Cleveland, Ohio, USA; 3 Infection Biology Program, Lerner Research Institute, Cleveland Clinic, Cleveland, Ohio, USA; Mayo Clinic Minnesota, Rochester, Minnesota, USA

**Keywords:** bacteriology, phenotypic identification, MALDI, gram-positive bacteria

## PHOTO QUIZ

**Fig 1:**
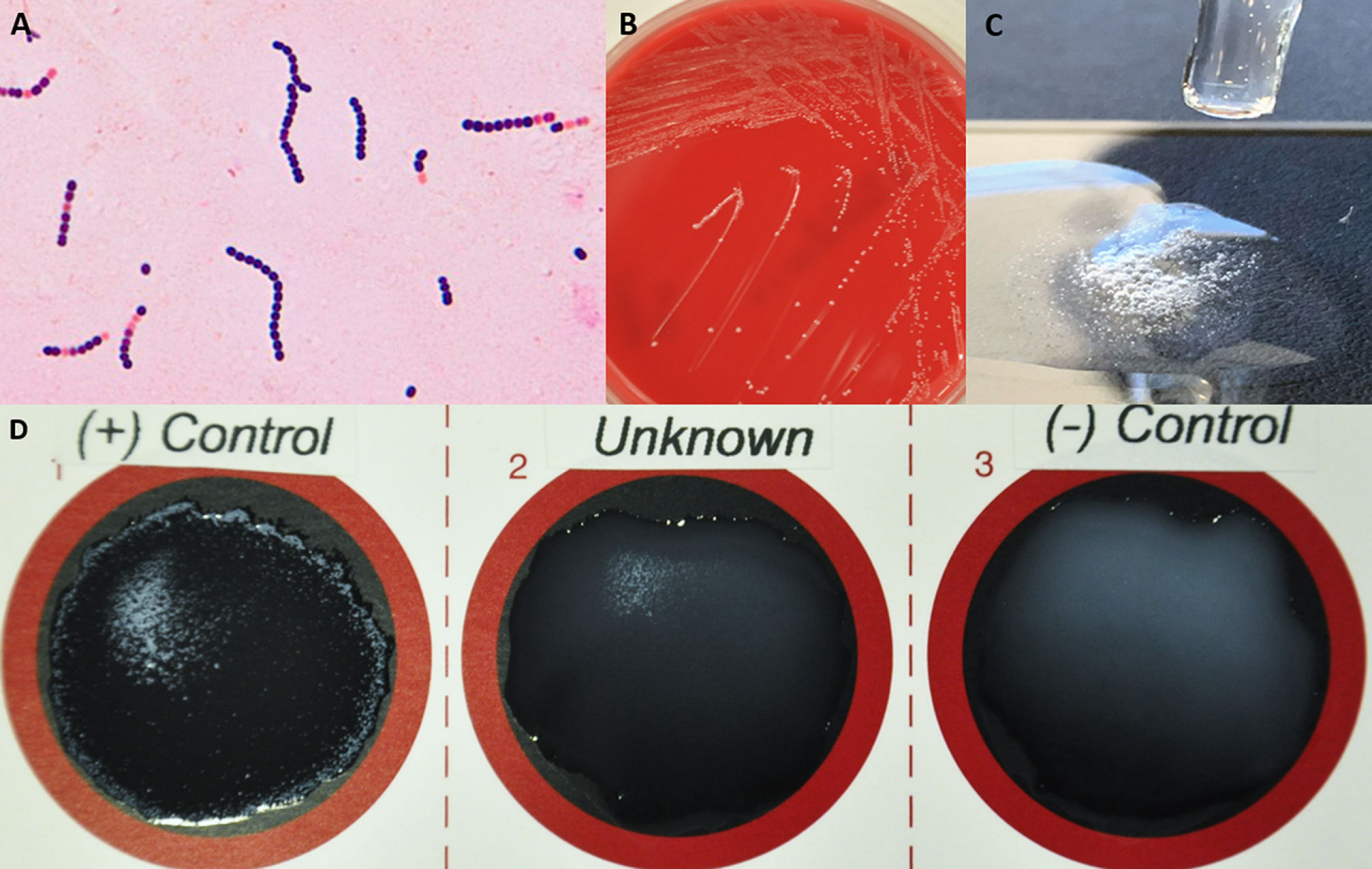
Gram stain of the blood culture bottle (**A**); colonies on sheep blood agar after 48-hour incubation (**B**); reaction when the colony was exposed to hydrogen peroxide (**C**); latex agglutination testing with the Lancefield group B reagent showing positive control, unknown patient specimen, and negative control (**D**).

A 55-year-old patient with a past medical history of diabetes, hypertension, and chronic kidney disease presents with malaise and fever for approximately 1 week. On physical examination, he was found to have a painful lower extremity, and non-healing ulcer present for approximately a month. The ulcer is 3.0 cm in diameter and ulcerated with a hyperemic border. He has two dogs and a pet fish. No significant travel or vocational history is noted. Blood cultures are collected and submitted to the microbiology laboratory. Growth was detected in the aerobic and anaerobic blood culture bottles after 16-hour incubation. A representative Gram stain of the blood cultures is pictured (Fig. 1A). Subcultured colonies were visible on the sheep blood agar (Fig. 1B: 48-hour incubation); there was no growth on MacConkey agar. Bubbles formed when the colony was exposed to hydrogen peroxide (Fig. 1C). Latex agglutination testing with the Lancefield group B reagent produced a weak reaction showing fine clumping (Fig. 1D). The VEREGENE System (Luminex) Gram-positive panel was negative. What is the diagnosis?

